# The role of angiotensin I converting enzyme insertion/deletion polymorphism in the severity and outcomes of COVID-19 patients

**DOI:** 10.3389/fgene.2022.1035796

**Published:** 2022-11-29

**Authors:** Mitra Rezaei, Hadiseh Mohammadpour, Mahya Eftekhari, Mihan Pourabdollah, Farinaz Nasr Azadani, Payam Tabarsi, Majid Marjani, Seyed Ali Ziai

**Affiliations:** ^1^ Department of Pathology, School of Medicine, Shahid Beheshti University of Medical Sciences, Tehran, Iran; ^2^ Clinical Tuberculosis and Epidemiology Research Center, National Research Institute of Tuberculosis and Lung Diseases (NRITLD), Shahid Beheshti University of Medical Sciences, Tehran, Iran; ^3^ Dental Research Center, Dentistry Research Institute, Tehran University of Medical Sciences, Tehran, Iran; ^4^ School of Medicine, Shahid Beheshti University of Medical Sciences, Tehran, Iran; ^5^ Chronic Respiratory Diseases Research Center, National Research Institute of Tuberculosis and Lung Diseases (NRITLD), Shahid Beheshti University of Medical Science, Tehran, Iran; ^6^ Department of Biology, Islamic Azad University, Damghan, Iran; ^7^ School of Medicine, Department of Pharmacology, Shahid Beheshti University of Medical Sciences, Tehran, Iran

**Keywords:** COVID-19, angiotensin I converting enzyme, insertion/deletion polymorphism, SARS-CoV-2, peptidyl dipeptidase A, ACE, angiotensin-converting enzyme, polymorphism

## Abstract

The pandemic of coronavirus disease in 2019 has led to a global crisis. COVID-19 shows distinct clinical manifestations of the severity of symptoms. Numerous patients with no associated risk factors demonstrate acute respiratory distress syndrome (ARDS). The role of genetic factors in determining the severity and outcome of the disease remains unresolved. The purpose of this study was to see if a correlation exists between Angiotensin I Converting Enzyme (*ACE*) insertion/deletion (I/D) polymorphism and the severity of COVID-19 patients’ symptoms. 120 COVID-19 patients admitted to Masih Daneshvari Hospital in Tehran with their consent to participate entered the study. Based on the World Health Organization classification, patients were divided into moderate and severe groups, which were primarily affected by O_2_ saturation levels. The effects of the patients’ *ACE* insertion/deletion polymorphism, background disease, Angiotensin receptor blocker (ARB) drug consumption, and demographic parameters on the severity risk were calculated statistically. The *ACE* D allele was associated with an increased risk of disease severity (OR = 6.766, *p* = 0.012), but had no effect on mortality.

## Introduction

COVID-19 cases show distinct clinical manifestations, most of which showing no symptoms or mild symptoms (Wu and McGoogan 2020), whereas a small proportion experience a severe form of disease resulting in severe acute respiratory syndrome. This condition is linked to high death rate (Grasselli, Zangrillo et al., 2020; Richardson, Hirsch et al., 2020). Adverse outcome is associated with some demographic characteristics, such as male gender (Zheng, Peng et al., 2020), age over 65 (Zheng, Peng et al., 2020), and being African-American (Doumas, Patoulias et al., 2020). Poor outcome is also associated with particular comorbidities, such as diabetes (Rod, Oviedo-Trespalacios et al., 2020), hypertension (Lippi, Wong et al., 2020), and body mass index (BMI) of 30 and above (Hussain, Mahawar et al., 2020), which is defined as obesity. Despite this, numerous patients without these characteristics have demonstrated acute respiratory distress syndrome (ARDS) (Zheng and Cao 2020). In the interim, the role of genetic factors in determining the severity and outcome of the disease remains unanswered (Ghafouri-Fard, Noroozi et al., 2020). Furthermore, there are many and varied factors involved in this process, such as age, environmental impacts, and the aforementioned risk factors. Therefore, further investigations are needed to assess the role of genetic susceptibility.

The novel coronavirus 2019 enters the host cells *via* angiotensin-converting enzyme 2 (*ACE*2) receptor. *ACE*2 is also a component of the renin-angiotensin (RAS) system and has a close interplay with *ACE* (Wu, Deng et al., 2020; Zhou, Yang et al., 2020). SARS-CoV epidemic began in 2002. SARS-CoV-2 and SARS-CoV have similar pathogenesis routes as they both bind to *ACE*2 to enter the host cells (Shi, Wang et al., 2020). In the case of SARS-CoV infection, as the virus binds to the *ACE*2 receptor, it can enter the cell and consequently causes down regulation of *ACE*2 (Ni, Yang et al., 2020). These processes are repeated in COVID-19 infection, indicating the call for a more comprehensive investigation of the role of RAS modulation in COVID-19 patients (Zheng and Cao 2020).

**FIGURE 1:**
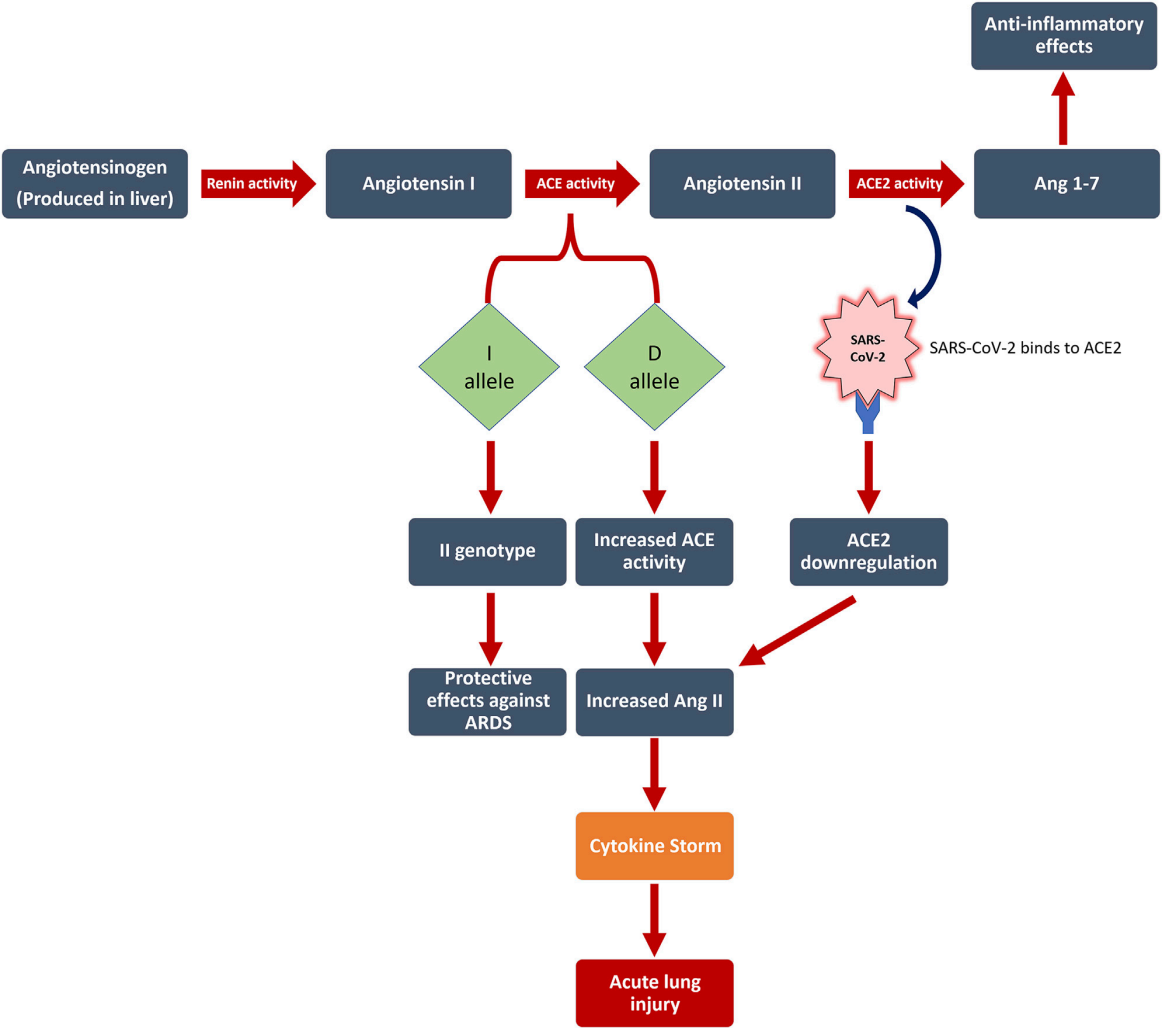
The interplay between RAS, Ang II levels, and SARS-CoV-2 infection is illustrated in this figure. Numerous patients with no associated risk factors demonstrate acute respiratory distress syndrome (ARDS). The role of genetic factors in determining the severity and outcome of COVID-19 disease remains unresolved. We discovered that the Angiotensin I Converting Enzyme (*ACE*) insertion/deletion (I/D) polymorphism affects the severity of patients’ symptoms, with the D allele increasing the risk of disease severity by 6.8 (*p* = 0.012), but having no effect on mortality. ACE: Angiotensin I Converting Enzyme; ACE2: Angiotensin-Converting Enzyme two; Ang 1–7: Angiotensin 1–7; D allele: Deletion allele; I allele: Insertion allele; ARDS: acute respiratory distress syndrome; SARS-CoV-2: severe acute respiratory syndrome coronavirus 2.

Angiotensin I is transformed to angiotensin II (Ang II) through *ACE* activity (Baudin 2002), after which Ang II is converted to Ang-(1–7) *via ACE*2 activity (Donoghue, Hsieh et al., 2000; Tipnis, Hooper et al., 2000). In the context of lowered *ACE*2 expression or raised *ACE* activity that happens in SARS-CoV-2 infection, an unrestricted level of Ang II is induced which, in turn, may bring about acute lung injury through several processes (Zheng and Cao 2020), (Figure 1).

Unrestricted levels of Ang II may have disruptive effects related to lung injury, and yet Ang II is produced by *ACE* activity. Thus, it can be concluded that *ACE* activity levels may also have an impact.

*ACE* is an enzyme, which can be found profusely in neuroepithelial, epithelial, and endothelial cells (Ziai, Salehian et al., 2004). *ACE* has kinase activity and is a zinc-dependent metallopeptidase (Mohammadpour; Ziai et al., 2020). *ACE* has a main role in regulation of blood pressure as well (Kouchmeshky, Jameie et al., 2012). The *ACE* gene is located on the 17q23 locus as assigned by Mattei et al., in 1989 (Mattei, Hubert et al., 1989). The polymorphism of the *ACE* gene is defined by the deletion (D) or insertion (I) of the 287-bp-long Alu element in the *ACE* gene, specifically in intron 16. The D allele is associated to the elevated activity of the *ACE* (Zheng and Cao 2020). Altogether, it is suggested that the polymorphism of *ACE* gene may have a prominent role to play in determining the clinical outcomes or severity of COVID-19 cases.

In the present study, we aim to identify if *ACE* insertion/deletion polymorphism plays a role in mortality and severity of patients’ symptoms.

## Materials and methods

This study was approved by the Ethics Committee of Shahid Beheshti University of Medical Sciences, Tehran, Iran, with the code IR. SBMU.MSP.REC.1399.519. All the performed experiments were in compliance with the relevant guidelines.

**1) Study cohorts:** Of the patients admitted to Masih Daneshvari Hospital in Tehran, 120 patients who tested positive for the COVID-19 polymerase chain reaction (PCR) test entered the study with their informed consent to participate. Demographic and anthropometric data and medical history of patients were obtained and recorded over the course of history taking. Recorded items included sex, age, existing comorbidities, such as diabetes mellitus, cardiovascular diseases, respiratory diseases, hypertension (systolic pressure ≥130 mm Hg or diastolic pressure ≥80 mm Hg), high BMI -in this study defined as BMI ≥30, which includes obese category according to Centers for Disease Control and Prevention (CDC), ((CDC) 2020), smoking and use of certain medications such as angiotensin II receptor blockers (ARBs), and *ACE* inhibitors (*ACE*I). Finally, the ultimate outcome of patients (recovery or death) was recorded.

According to World Health Organization (WHO) classification of COVID-19 severity, patients were divided into four groups based on the severity level of their condition. Mild condition is defined as patients meeting case definition criteria showing symptoms of COVID-19 infection, such as sore throat, cough, shortness of breath, fever, malaise, muscular pain, headache, nausea, vomiting, loss of smell and taste, diarrhea, anorexia, and nasal congestion but with no signs of hypoxia or viral pneumonia. Moderate condition is defined as having clinical signs of pneumonia, including dyspnea, tachypnea, cough, and fever, but not showing signs of severe form of pneumonia, including oxygen saturation (SpO_2_) ≥ 90% on room air. Severe condition is defined as manifestation of clinical signs of pneumonia, such as dyspnea, cough, fever in addition to one of the following criteria: severe respiratory distress, respiratory rate >30 per minute, or SpO_2_ < 90% on room air. Critical condition is defined by having the criteria for ARDS, septic shock, sepsis, or other settings that would generally necessitate providing the life-saving treatments, such as invasive or non-invasive mechanical ventilation or vasopressor administration (World Health Organization 2022). Since individuals with mild disease were managed in out-patient settings, we did not have access to them within the in-patient admissions. Patients with moderate disease were assigned to the moderate group, while the severe and critical groups were assigned to the severe group.

**2) DNA extraction:** In order to determine the polymorphism of the *ACE* gene (II/ID/DD) in patients, 5 ml of blood was collected from each participant. Blood samples were centrifuged at 2500xg for 15 min. The supernatant plasma layer was then discarded and for every 1 ml of blood, 3 ml of red blood cell (RBC) lysis solution was added to the remaining part. BioFact™ Genomic DNA Prep Kit (South Korea) was used for DNA extraction step. The purity and quantity of the extracted DNA was finally measured by the NanoDrop 1000 device and the data for each sample were recorded.

**3) Genotyping**: Gap-PCR method was conducted. After DNA extraction, Gap-PCR was performed to determine the genotype of I/D polymorphism of the *ACE* gene. Taq DNA Polymerase Master Mix (Ampliqon, Denmark) was used for carrying out the PCR. The reaction components of the PCR consisted of 3 μl of DNA sample, 10 μl of master mix (X2), 1 μl of forward and reverse primer solution, and 6 μl of DNase-RNase free water. The reaction was performed with regard to PCR thermal profile. Afterwards, agarose gel electrophoresis was used to identify the existing alleles and the results were examined under UV-transilluminators. For this step 6x loading dye-MM2121 (Sinaclon, Iran), and Ladder 50 bp (ready to use) 50-1500 bp and Ladder 100bp Plus (ready to use) 100-3000 bp (Sinaclon, Iran) were used for dyeing, and as the markers respectively. The primers’ sequence was as follows:

**Table:** 

Forward primer	5′- CTG​GAG​ACC​ACT​CCC​ATC​CTT​TCT-3′
Reverse primer	5′- GAT​GTG​GCC​ATC​ACA​TTC​GTC​AGA​T-3′

**4) Statistical analysis**: For risk estimates (odds ratio (OR)), the Pearson’s Chi-square test was used. Scale variables were tested for normal distribution using Kolmogorov–Smirnov, and independent t-tests were used to compare the mean of normal distributed variables. The Mann Whitney U test was used to compare non-parametric variables. For modeling and calculating adjusted OR, binary logistic regression (Enter method) was used. In this study, IBM SPSS software version 16 was used.

## Results

**FIGURE 2:**
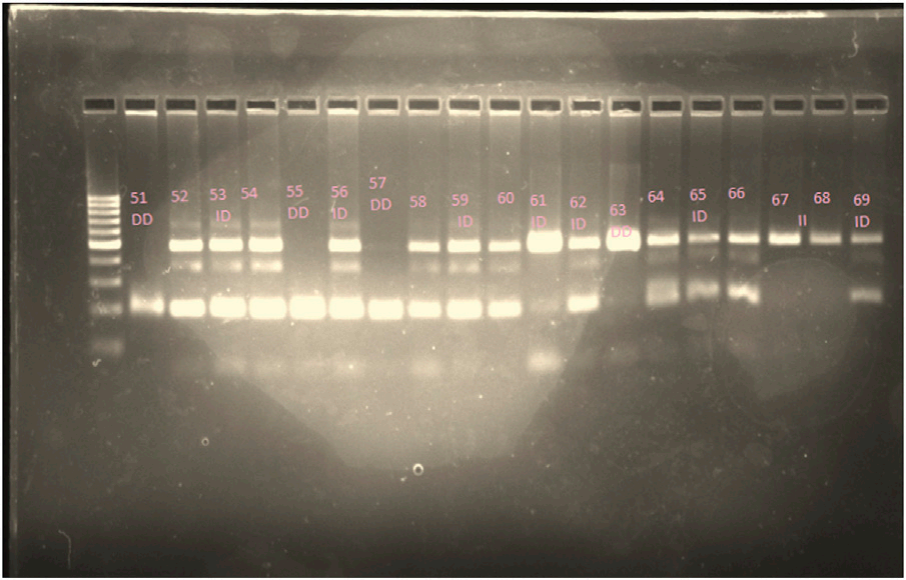
Gel electrophoresis showing genotypes of 19 patients. In this study 120 patients were genotyped and 24, 82, and 14 patients were identified with DD, ID, and II genotypes respectively. *ACE* polymorphism genotypes (DD/ID/II) of patients 51–69 are indicated in this figure.

In this study, 120 COVID-19 patients (61 males, 59 females) were studied, with a mean age of 52.1 ± 18.94 years (range: 14–93 years), a mean height of 1.66 ± 0.088 m (range: 1.47–1.83), and a mean BMI of 28.1 ± 6.37 (range: 15.79–49.95). 7 (5.8%) of the studied patients were smokers, 23 (19.2%) had diabetes mellitus (DM), 14 (11.7%) had cardiovascular disease (CVD), 6 (5.0%) had respiratory disease (RD), 38 (31.7%) had hypertension (HTN), and 25 (20.8%) were taking ARB drugs. Of all patients, 74 (61%) were admitted to the intensive care unit (ICU). In our study, 26, 21, and 73 patients had moderate, severe, and critical form of the disease, respectively. We genotyped all 120 patients to determine the I/D polymorphism of the *ACE* gene (gel electrophoresis of 19 patients is shown in Figure 2). In this study the genotypes of 120 patients were determined and 24, 82, and 14 patients were identified with DD, ID, and II genotypes respectively.

Patients carrying the *ACE* D allele had a higher risk of disease severity (OR = 6.519, *p* = 0.001), and it was an independent variable in disease severity modeling using the logistic regression method (OR = 6.766, *p* = 0.012), (Table 1).

**TABLE 1:** Distribution of COVID-19 patients parameters based on disease severity. Adjusted odds ratio was calculated based on binary logistic regression analysis.

			Severity		OR	95% CI	p-value*	Adjusted OR	p-value
			Moderate n = 26	Severe n = 94					
Sex	Male	N	11	50	0.645	0.268–1.551	0.326	0.670	0.489
		%	42.3%	53.2%					
	Female	N	15	44					
		%	57.7%	46.8%					
Diabetes	No	N	23	74	2.072	0.564–7.607	0.264	2.580	0.259
		%	88.5%	78.7%					
	Yes	N	3	20					
		%	11.5%	21.3%					
Cardiovascular disease	No	N	24	82	1.756	0.367–8.395	0.476	0.391	0.356
		%	92.3%	87.2%					
	Yes	N	2	12					
		%	7.7%	12.8%					
Respiratory disease	No	N	25	89	1.404	0.157–12.579	0.760	5.7 E+08	0.999
		%	96.2%	94.7%					
	Yes	N	1	5					
		%	3.8%	5.3%					
Hypertension	No	N	21	61	2.272	0.785–6.580	0.124	0.554	0.540
		%	80.8%	64.9%					
	Yes	N	5	33					
		%	19.2%	35.1%					
High BMI	No	N	15	62	1.306	0.431–3.958	0.636	1.769	0.383
		%	75.0%	69.7%					
	Yes	N	5	27					
		%	25.0%	30.3%					
Smoking	No	N	25	88	1.705	0.196–14.826	0.625	0.622	0.699
		%	96.2%	93.6%					
	Yes	N	1	6					
		%	3.8%	6.4%					
ARB drugs	No	N	23	72	2.343	0.642–8.548	0.187	1.129	0.898
		%	88.5%	76.6%					
	Yes	N	3	22					
		%	11.5%	23.4%					
D allele (*ACE*)	Negative	N	8	6	6.519	2.016–21.080	0.001	6.766	0.012
		%	30.8%	6.4%					
	Positive	N	18	88					
		%	69.2%	93.6%					
I allele (*ACE*)	Negative	N	3	21	0.453	0.124–1.659	0.223	0.877	0.857
		%	11.5%	22.3%					
	Positive	N	23	73					
		%	88.5%	77.7%					

N, number of patients; OR, odds ratio; CI, confidence interval.

*Pearson’s Chi-Square test.

**FIGURE 3:**
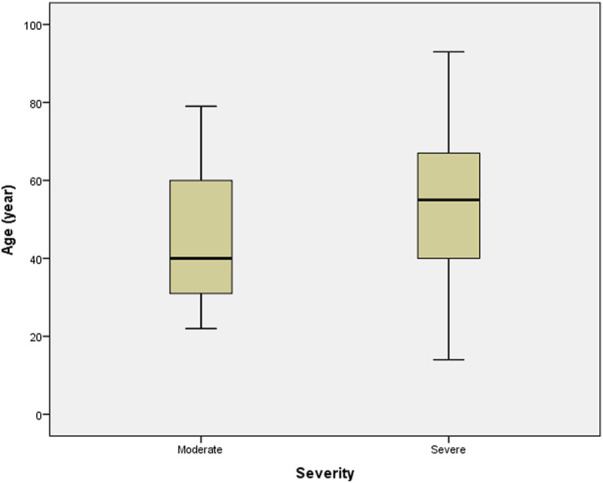
Distribution of the patients’ age among severity of the disease groups. Boxplots show the median and interquartile range, and whiskers represent the extreme cases of individual variables. The mean age of individuals in severe disease group was significantly higher than that of in the moderate disease group (*p* = 0.016).

Patients with severe disease had a significantly higher mean age (54.32 ± 18.96 years) than those in the moderate group (44.23 ± 16.91 years) (Independent t-test, *p* = 0.016), Figure 3. Patients with moderate and severe disease had mean BMIs of 27.81 ± 6.09 and 28.28 ± 6.46, respectively (non-significant) which is shown in Table 2.

**TABLE 2:** Distribution of age and BMI among the disease groups (severe and moderate). Data show mean value (standard deviation) and *p* values were calculated based on two independent sample student *t* test.

	Moderate	Severe	p-value
Age (year)	44.23 (16.91)	54.32 (18.96)	0.016
BMI	27.81 (6.09)	28.28 (6.46)	0.769

Table 3 shows that while background disease (DM, HTN, CVD, and RD) did not increase the risk of COVID-19 severity, O_2_ saturation did. A significantly higher proportion of patients with moderate than severe disease have the *ACE* II polymorphism (OR = 0.153; *p* = 0.002).

**TABLE 3:** Analysis of the effects of background disease (the sum of the disease mentioned in Table 1), O_2_ saturation, and *ACE* polymorphism on disease severity.

			Severity		OR	95% CI	p-value*
			Moderate n = 26	Severe n = 94			
Disease background	No	N	16	42	1.981	0.815–4.817	0.128
		%	61.5%	44.7%			
	Yes	N	10	52			
		%	38.5%	55.3%			
O_2_≤90	No	N	24	27	28.889	6.377–130.862	1.052 × 10^−8^
		%	92.3%	29.3%			
	Yes	N	2	65			
		%	7.7%	70.7%			
O_2_≤93	No	N	21	11	30.927	9.686–98.752	3.194 × 10^−12^
		%	80.8%	12.0%			
	Yes	N	5	81			
		%	19.2%	88.0%			
*ACE* II	Negative	N	18	88	0.153	0.047–0.496	0.001
		%	69.2%	93.6%			
	Positive	N	8	6			
		%	30.8%	6.4%			
*ACE* ID	Negative	N	11	27	1.820	0.742–4.463	0.188
		%	42.3%	28.7%			
	Positive	N	15	67			
		%	57.7%	71.3%			
*ACE* DD	Negative	N	23	73	2.205	0.603–8.071	0.223
		%	88.5%	77.7%			
	Positive	N	3	21			
		%	11.5%	22.3%			

N: number of patients; OR: odds ratio; CI: confidence interval.

* Pearson’s Chi-Square test.

Based on the patient outcome (dead or recovered), our findings were consistent with others in that background disease increased the risk of mortality (OR = 3.469, *p* = 0.008), with hypertension being a significant risk factor (OR = 3.096, *p* = 0.01). The *ACE* polymorphism, on the other hand, had no effect on mortality. Recovered patients were younger than deceased patients (49.51 ± 18.31 vs. 61.19 ± 18.55 *p* = 0.004).

## Discussion

Two factors that play a key role in RAS modulation, are *ACE* and *ACE*2 (Crowley, Gurley et al., 2005). Since the equilibrium between *ACE* and *ACE*2 is crucial in homeostasis of RAS, it seems that *ACE* and *ACE*2 have a mutual interaction to achieve this (Zheng and Cao 2020).

Unrestricted levels of Ang II can lead to acute lung injury through several processes (Zheng and Cao 2020). These include provoking fibrosis (Marshall, Gohlke et al., 2004), promoting apoptosis of alveolar epithelial cells and endothelial cells (Dimmeler, Rippmann et al., 1997; Wang, Feng et al., 2000), increasing vasculature permeability and subsequent vasoconstriction (Zheng and Cao 2020), and finally, augmentation of pro-inflammatory cytokines, such as interleukin 6 (IL-6) and IL-8 (Suzuki, Ruiz-Ortega et al., 2003). IL-6 is a main factor of the cytokine storm and plays an important role in the severity of COVID-19 patients (Hadjadj, Yatim et al., 2020; Mahmoudi; Rezaei et al., 2020).

Our study found that in the severity outcome, the *ACE* D allele and older age are risk factors for disease severity, but in the mortality outcome, background disease, specifically hypertension, and older age are risk factors.

Studies on animal models and humans advocate the role of raised levels of Ang II caused by *ACE* and *ACE*2 imbalances in pathogenesis of acute lung injury (Zheng and Cao 2020).

In a cohort study of COVID-19 patients, plasma levels of Ang II were found to be meaningfully higher than the healthy control group. In addition, Ang II levels showed a linear correlation with the severity of the injury and the virus burden (Liu, Yang et al., 2020). This piece of evidence suggests that SARS-CoV-2 infection results in increased amount of *ACE* molecule or activity. In a study on a rat model, high-volume ventilation was employed to foster lung injury, and subsequently, aggravated lung injury which correlated with lung Ang II overproduction, was observed (Jerng, Hsu et al., 2007). As for influenza, the animal model manifested severe lung injury, seemingly correlated with decreased expression of *ACE*2 (Yang, Gu et al., 2014). In another mouse model, SARS-CoV binding to *ACE*2 *in vivo* showed declined expression of *ACE*2 and augmented acute lung injury (Kuba, Imai et al., 2005). However, some studies have demonstrated different results. Biancatelli et al. have shown that exposure of subunit one of the SARS-CoV-2 spike protein (S1SP) to in-house harvested human lung microvascular endothelial cells does not change *ACE2* expression significantly (Colunga Biancatelli, Solopov et al., 2022). Another study conducted by Solopov et al. on homogenates of lung tissue of mice which were on alcohol diet *versus* those on control diet, showed alcohol increased *ACE2* expression in alcohol-consumption group but *ACE2* expression was not further affected by S1SP exposure (Solopov, Colunga Biancatelli et al., 2022).

A large body of evidence exists that supports the link between *ACE* genotype and severity of ARDS outcomes (Marshall, Webb et al., 2002; Pati, Mahto et al., 2020; Sarangarajan, Winn et al., 2020; Yamamoto, Ariumi et al., 2020). In individuals with DD genotype, the average levels of *ACE* activity were nearly twice as high as the II genotype. This insertion/deletion (I/D) polymorphism constitutes about half of the diversity of *ACE* tissue and serum levels observed among individuals (Zheng and Cao 2020).

Our results showed that a higher proportion of patients with severe disease had the D allele. In a meta-analysis study in 2015, it was observed that I/D polymorphism could be a risk factor if the genotype was DD, and the DD genotype showed an elevated risk in ARDS development compared to ID and II genotypes (Deng, Zhang et al., 2015). Another study by Tsantes et al. on patients with acute lung injury and ARDS demonstrated that D allele strongly correlated with higher death rates, and I/D polymorphism seemed to impact the serum *ACE* levels associated with prognosis in these patients (Tsantes, Kopterides et al., 2013). Another study showed the association of DD genotype with mortality in the ARDS population. In addition, patients with II polymorphism were associated with markedly better rates of survival (Ziai et al., 2004; Jerng, Yu et al., 2006; Rezaei et al., 2020). In a study conducted by Itoyama et al., in severe acute respiratory syndrome (SARS) patients, it was shown that the D allele was higher in frequency in the hypoxemic group compared to the non-hypoxemic group (Itoyama, Keicho et al., 2004). In a study by Bellone et al. *ACE* DD polymorphism was reported to be associated with COVID-19 mortality rate (Bellone and Calvisi 2020). Similar results have also been reported in some other studies conducted on patients with COVID-19. In the study by Pati et al. it was revealed that D allele was associated with mortality rate of COVID-19 patients in Asians (Pati, Mahto et al., 2020). The DD polymorphism has been shown to affect the outcomes and increase the severity of the disease, as reported in the study by Sarangarajan et al., (Sarangarajan, Winn et al., 2020). Furthermore, Yamamoto et al. reported that *ACE* II genotype correlated negatively with number of COVID-19 cases and COVID-19-related mortalities (Yamamoto, Ariumi et al., 2020). A meta-analysis also concluded that *ACE* I/D polymorphism can be a beneficial marker for acute lung injury/ARDS prognosis and the genotypes may be used to treat acute lung injury/ARDS in COVID-19 patients. The mentioned study also showed that significant connection existed between risk of acute lung injury/ARDS and *ACE* polymorphism in children and Caucasian population, in addition to in Asian population in analysis of mortality (Pabalan, Tharabenjasin et al., 2021). A similar result was stated by another study regarding the prognostic value of the *ACE* genotype, and its impact on results of COVID-19 treatment (Verma, Abbas et al., 2021). A recent study reported close association between *ACE* polymorphism and COVID-19 infection severity, in which the II genotype showed protective effects upon developing the severe form of COVID-19 (Gunal, Sezer et al., 2021).

In our results, allele D was not associated with the outcome (recovery or death). In the study of Chan et al., it was reported that no association existed between *ACE* polymorphism and undesirable outcomes after SARS infection (Chan, Tang et al., 2005). A meta-analysis also indicated that no association exists between *ACE* polymorphism and susceptibility to ARDS or acute lung injury, but a connection is present between *ACE* polymorphism and mortality among Asian patients with ARDS (Matsuda, Kishi et al., 2012). Also, another study reported that *ACE* polymorphism and ARDS were not associated, however, plasma Ang II levels were higher in the ARDS group of D-allele carriers, but not in the control group, and the frequency of severe hypoxemia was less in carriers of D-allele (Cruces, Díaz et al., 2012). One study on COVID-19 showed a positive correlation between frequency ratio of I/D alleles and rate of recovery, but no difference in regard to the mortality rate (Hatami, Ahi et al., 2020).

The limitations on this study were the lack of group of patients with mild severity of the disease *versus* moderate and severe groups which was due to the out-patient management of individuals with mild disease and we did not have access to them within the in-patient admissions.

## Conclusion

We concluded that *ACE* II genotype was significantly higher in proportion within the moderate disease group compared to the severe disease group, and that the D allele was associated with a higher risk of disease severity but not mortality.

## Data Availability

The datasets presented in this article are not readily available because of privacy and ethical issues. Requests to access the datasets should be directed to Seyed Ali Ziai, aliziai@sbmu.ac.ir.
